# QTL Mapping of Genome Regions Controlling Manganese Uptake in Lentil Seed

**DOI:** 10.1534/g3.118.200259

**Published:** 2018-03-27

**Authors:** Duygu Ates, Secil Aldemir, Bulent Yagmur, Abdullah Kahraman, Hakan Ozkan, Albert Vandenberg, Muhammed Bahattin Tanyolac

**Affiliations:** * Department of Bioengineering, Faculty of Engineering, Ege University, Bornova, Izmir, Turkey; † Department of Field Crops, Faculty of Agriculture, Harran University, Sanliurfa, Turkey; ‡ Department of Field Crops, Faculty of Agriculture, Cukurova University, Adana, Turkey; § Crop Development Centre, University of Saskatchewan, Saskatoon, Saskatchewan, Canada

**Keywords:** DArT, SNP, QTL mapping, manganese, lentil, Genome Report

## Abstract

This study evaluated Mn concentration in the seeds of 120 RILs of lentil developed from the cross “CDC Redberry” × “ILL7502”. Micronutrient analysis using atomic absorption spectrometry indicated mean seed manganese (Mn) concentrations ranging from 8.5 to 26.8 mg/kg, based on replicated field trials grown at three locations in Turkey in 2012 and 2013. A linkage map of lentil was constructed and consisted of seven linkage groups with 5,385 DNA markers. The total map length was 973.1 cM, with an average distance between markers of 0.18 cM. A total of 6 QTL for Mn concentration were identified using composite interval mapping (CIM). All QTL were statistically significant and explained 15.3–24.1% of the phenotypic variation, with LOD scores ranging from 3.00 to 4.42. The high-density genetic map reported in this study will increase fundamental knowledge of the genome structure of lentil, and will be the basis for the development of micronutrient-enriched lentil genotypes to support biofortification efforts.

Lentil originated in southwestern Asia and its seeds have been consumed since prehistoric times. The origin of cultivated lentil is the Near East Arc and Asia Minor (Muehlbauer and McPhee 2005). Lentils are grown throughout the world, with production from Canada, India, Turkey, and Australia providing most of the world’s supply (Ford *et al.* 2007). Lentil seeds are an excellent source of manganese (Mn), yet deficiency of this micronutrient affects 35% of children worldwide (Qaim *et al.* 2007).

Mn is an essential micronutrient with a recommended daily intake of 0.7 to 22.0 mg for adults (Santos *et al.* 2004). Mn health benefits include development of normal bone structure, metabolism of bones, and promotion of the necessary enzymes for bone health (Price *et al.* 2012). Mn acts as a co-enzyme to assist metabolic activity in the body (Finkelstein *et al.* 2007) and is involved in the synthesis of RNA, DNA, and proteins (Xu *et al.* 2002). Other health benefits associated with adequate Mn intake include the formation of joint tissues (Kannus 2000), proper functioning of the thyroid gland and sex hormones (Soldin and Aschner 2007), metabolism of carbohydrates and fats, and regulation of blood sugar levels (Van den Berghe 2004). Adequate Mn is important for brain function (Takeda 2003) and nervous system activity throughout the body (Erikson and Aschner 2003). Symptoms of Mn deficiency include skeletal abnormality, heart ailments (Witte *et al.* 2001), high cholesterol (Davis *et al.* 1990), muscular contraction, poor visual and auditory function, high blood pressure, tremors and shivers (Komaki *et al.* 1999), severe memory loss, and bone malformation (Bourre 2006).

Plant-based dietary habits are the leading cause of Mn deficiency due to the low micronutrient density of foods, a problem that can be addressed by biofortification, dietary diversification, or supplementation. For the past two decades, researchers have focused on biofortification strategies (Zhu *et al.* 2007; Mayer *et al.* 2008; Blancquaert *et al.* 2014; Zou *et al.* 2014; Ates *et al.* 2016; Nakandalage *et al.* 2016; Aldemir *et al.* 2017; Garcia-Casal *et al.* 2017; Giuliano 2017; Sharma *et al.* 2017). Biofortification is a means of increasing the daily micronutrient consumption of individuals who suffer from micronutrient malnutrition (Bouis 2003). The goal of biofortification is to increase the concentration of micronutrients in the edible part of crop plants through plant breeding (Carvalho and Vasconcelos 2013). Research on increasing the concentration of minerals in seeds has largely focused on crops such as maize (Oikeh *et al.* 2003; Gupta *et al.* 2015; Prasad *et al.* 2015), rice (Gregorio *et al.* 2000; Gupta *et al.* 2015; Prasad *et al.* 2015; Nakandalage *et al.* 2016), wheat (Monasterio and Graham 2000; Garvin *et al.* 2006; Gupta *et al.* 2015; Prasad *et al.* 2015), barley (Ma *et al.* 2004; Rodrigo *et al.* 2013), and lentil (Ates *et al.* 2016; Aldemir *et al.* 2017). Identifying the quantitative trait loci (QTL) that control the concentration of Mn in lentils would aid in the development of biofortified cultivars through the use of closely linked molecular markers, which would allow breeders to screen and select for micronutrient dense genotypes. QTL controlling Mn uptake and identified using QTL analysis have been published for cabbage (Wu *et al.* 2008), *Lotus japonicus* (Klein and Grusak 2009), clover (Sankaran *et al.* 2009), canola (Ding *et al.* 2010), and Arabidopsis (Willems *et al.* 2010). To date, however, no QTL controlling the concentration of Mn in lentil seeds have been identified. The objectives of this study were to (*i*) determine the Mn concentration of lentil seeds from recombinant inbred lines (RILs), (*ii*) calculate genetic variation of Mn concentration among RILs, locations, and years, and (*iii*) identify QTL controlling Mn concentration in lentil seeds.

## Materials And Methods

### Soil analysis

Soil samples were collected from experimental fields in three locations in Turkey (Izmir, Adana, and Sanliurfa) to determine the physical and chemical properties of each soil. Soil pH analysis (Black 1965), total soluble salt analysis (Richards 1954), texture analysis (Bouyoucos 1955), organic matter analysis (Black 1965), CaCO_3_ analysis (Schlicting and Blume 1966), and macro- and micro-nutrient analysis (Bingham 1949; Pratt 1965; Lindsay and Norvell 1978) were carried out at the Department of Plant and Soil Science at Ege University in Izmir, Turkey.

### Plant materials

A population of 120 RILs was developed from the cross “CDC Redberry” (P1) × “ILL7502” (P2) and designated LR-8. P1 was developed from a cross made in 1997 between CDC breeding lines 1049F3 / 819-5R. Line 1049F3 was derived from the cross 567-16 / 545-8. Line 819-5R was derived from the cross 86-360 / (458-258G / (458-122 / C8L27- RC // Precoz) F2) F1 (Vandenberg *et al.* 2006). P2 is a lentil cultivar released in Bangladesh (Sarker *et al.* 1999). The LR-8 population was generated by advancing F_1_ plants from the simple cross to the F_2_ generation, and the RILs developed by single seed descent from the F_2_ to the F_7_ generation. The RILs were produced at the University of Saskatchewan, Canada where resources for genetic and genomic studies of lentil have been developed since 2001.

### Micronutrient analysis and heritability

The RILs were grown in 2 years (2012 and 2013) in three different locations in Turkey—Ege University Izmir (27°09’ E, 38°25’ N), Cukurova University in Adana (35°18’ E, 37°01’ N), and Harran University in Sanliurfa (38°46’ E, 37°08’ N)—and placed with three replications in a randomized complete block design (RCBD) with three factors (year, location, genotype) for micronutrient analysis. An atomic absortion spectrometer (AAS) (Varian, SpectrAA 220/ FS, California, USA) was used to estimate Mn concentrations in all seed samples. Samples were prepared for analysis as per a previous study (Kacar 1972). Seed samples (2 g) were first washed with tap water and then with pure water to remove surface contaminants. The washed seeds were dried in a hot air oven at 65°. The dried samples were ground using an analytic mill (IKA, A11, Staufen, Germany) and then each 2-g ground sample placed into a 150-mL flask to which 24 mL of 4:1 nitric:percholoric acid were added to decompose the samples. All procedures were performed with three replications. Spectrophometric readings for total Mn concentrations were converted to mg/kg concentration in seed (Kacar 1972; Kacar and Inal 2008). To confirm the accuracy of the AAS, standard Mn solutions (1.0, 2.0, 3.0, 4.0, 5.0 ppm) were analyzed to form a calibration curve (*r^2^* = 0.9999). Heritability (H) based on lentil population means was calculated with the formula H = [MS_among families_ – MS_year_*_family_]/MS_among families_, where MS is the mean square (Courtney *et al.* 2008).

### Variance analysis

Analysis of variance (ANOVA) was used to determine variation in Mn concentrations of the LR-8 RIL population grown in different years and locations using TOTEMSTAT software (Acikgoz *et al.* 2004). Genotypes were accepted as fixed while year and location were random. Variation of year (Y) × location (L), Y × genotype (G), L × G, and Y × L × G interactions were calculated and significance was accepted at the *P* ≤ 0.01 and ≤ 0.05 levels.

### DNA isolation

Young leaves from 4- to 6-week-old seedling of all lentil genotypes grown at the Izmir location were harvested and placed in labeled aluminum foil containers and then immediately placed in liquid nitrogen. Frozen leaf samples were kept in a deep freezer (-86°) until analysis. Genomic DNA from 120 RILs and the parents were extracted from frozen leaf tissue using the Fermentas DNA Isolation Kit (Thermo Scientific, Hanover, MD, USA). Purity of the DNA was confirmed on 1% agarose gel and the purified DNA then quantified with a Qubit2.0 Fluorometer (Life Technologies, US).

### DArT analysis

DArT analyses were carried out following Aldemir *et al.* (2017). The raw data for SNP discovery are presented as supplemental file 1 (File S1).

### Linkage mapping and QTL analysis

JoinMap4.0 software described by Van Ooijen and Voorrips (2004) was used for linkage mapping analysis. A maximum recombination frequency of 0.50 and the kosambi function were used as options in linkage mapping. Distorted markers were eliminated. MapQTL version 6.0 (Van Ooijen 2009) was used for QTL analysis. The effects and positions of QTL were determined following composite interval mapping (CIM). The significant threshold was calculated based on 1000 permutations at the *P* ≤ 0.01 and ≤ 0.05 levels (Van Ooijen and Voorrips 2004), and QTL that passed the threshold significance are reported. The proportion of observed phenotypic variation explained due to a particular QTL was estimated by the coefficient of determination (*R^2^*) using maximum likehood for CIM.

### Data Availability

File S1 contains SNP data. File S2 contains Mn phenotyping data.

## Results

### Soil properties

The physico-chemical properties of soil samples from Izmir, Adana, and Sanliurfa locations are presented in Table 1. Soil samples from all locations were slighty alkaline, non-saline, and calcareous. Soil from Adana had a loamy clay texture and soils from the other two locations had a loamy texture. The bioavailability of Mn by plants from soil is the degree to which an extractable solid-phase quantity is correlated with measured tissue concentration, which is called available Mn (Lindsay and Norvell, 1978). Available Mn contents were low for all three soils.

**Table 1:** Physico-chemical properties of soil samples from Izmir, Adana, and Sanliurfa

**Soil properties**	**Izmir**	**Adana**	**Sanliurfa**
**pH**	7.82	7.74	7.76
**Total salt** (%)	0.04	0.03	0.04
**CaCo_3_** (%)	29.4	48.0	34.5
**Organic matter** (%)	1.86	1.29	1.96
**Fine sand** (%)	50.24	44.24	44.24
**Silt** (%)	28.00	26.00	32.00
**Clay** (%)	21.76	29.76	23.76
**Texture**	Loamy	Loamy clay	Loamy
**Total N** (%)	0.06	0.05	0.05
**Available P** (mg/kg)	3.29	2.62	1.87
**Available K** (mg/kg)	417	116	485
**Available Ca** (mg/kg)	6,272	6,762	7,252
**Available Mg** (mg/kg)	554	170	430
**Available Na** (mg/kg)	220	307	20
**Available Fe** (mg/kg)	4.93	5.01	6.52
**Available Zn** (mg/kg)	0.73	0.45	1.05
**Available Cu** (mg/kg)	1.13	0.19	0.65
**Available Mn** (mg/kg)	5.30	4.61	8.07

### Mn concentration in seeds of the LR-8 population

**Figure 1:**
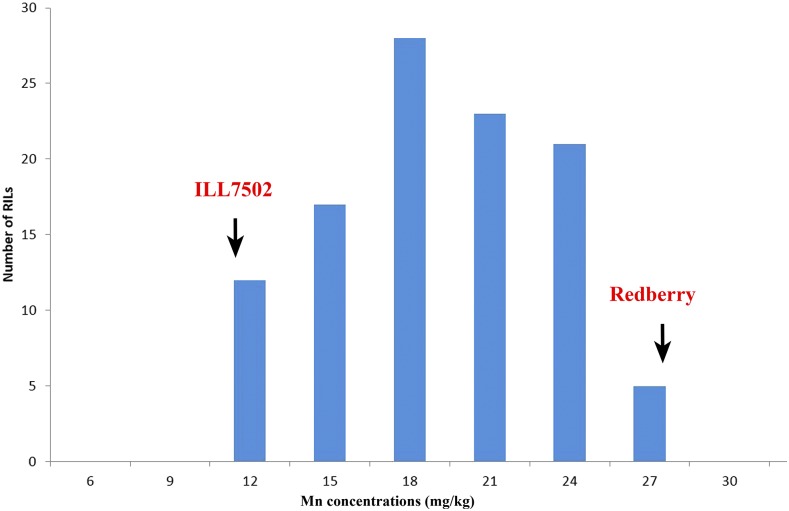
Frequency distribution of Mn concentration in lentil seeds of 120 RILs and their parents, averaged over three locations (Izmir, Adana, and Sanliurfa) and 2 years (2012 and 2013).

Mn concentrations in P1 and P2 of the LR-8 lentil population are shown in Table 2. The overall mean Mn concentration in seeds of the parents was 9.6 mg/kg for P2 and 27.6 mg/kg for P1. Mn concentrations in seeds of the RILs of the LR-8 population varied from 8.5 to 26.8 mg/kg with a mean of 17.6 mg/kg. The highest concentration of Mn was detected in RIL LR8-113. Heritability for Mn concentrations was detected as 0.76 and 0.74 for 2012 and 2013, respectively (Table 2). This means that Mn accumulation in the seed is affected by genetics rather than the environment. The frequency distribution of Mn concentrations in seeds of the LR-8 population as a mean across three locations and 2 years (Figure 1) shows that concentrations for the RIL population were also continuous but with a near to normal binomial distribution.

**Table 2:** Minimum, maximum, and mean Mn concentration in seeds of the LR-8 lentil population grown at Izmir, Adana, and Sanliurfa in 2012 and 2013

**Mn concentration (mg/kg)**							
**Location**	**Izmir**		**Adana**		**Sanliurfa**		**Mean**
**Year**	**2012**	**2013**	**2012**	**2013**	**2012**	**2013**	
**P1**	25.5	29.3	24.0	32.1	27.5	27.1	27.6
**P2**	7.7	8.8	9.5	10.6	8.0	12.7	9.6
**Minimum**	7.7	10.8	7.5	9.2	8.0	9.0	8.5
**Maximum**	27.8	31.9	27.5	26.3	28.5	25.1	26.8
**Mean**	17.8	20.4	17.5	17.3	17.8	17.3	17.6
**Mn heritability**	**2012**	**2013**					
	0.76	0.74					
**Std dev**	4.2	4.0					

Variance analysis showed that Mn concentration in seeds among RILs was significant at the *P* ≤ 0.01 level. It was also statistically significant among locations (Table 3). The effects of Y × L, Y × G, L × G, and Y × L × G interactions were statistically significant. Genotypes accumulated Mn in seed at different levels according to year and location.

**Table 3:** ANOVA for Mn concentrations in seeds of LR-8 lentil RILs grown at three different locations for two years

	**Mn concentration**				
**Source of Variation**	**df**	**Mean Square**	**F**	**F prob. 5%**	**F prob. 1%**
Block	2	4.1	2.4 ns	3.1	4.8
Year (Y)	1	55.7	31.6 **	3.9	6.9
Location (L)	2	14.1	8.0 **	3.1	4.8
Genoype (G)	119	306.0	173.4 **	1.4	1.5
Y × L	2	18.5	10.5 **	3.1	4.8
Y × G	119	3.1	1.8 **	1.4	1.5
L × G	238	4.0	2.3 **	1.3	1.4
Y × L × G	238	3.8	2.2 **	1.3	1.4
Error	1246	1.765			
General	1871				
	Coefficient of variation (CV)= 7.71%				

df: degree of freedom. ns: not significant.

** : Significant at *P* ≤ 0.01.

### Construction of linkage maps

Missing data and the data showing segregation distortion were filtered. After filtering, a total of 10,552 SNPs were developed through DArT. Among them, 5,385 SNPs could be mapped in the lentil genome. The LR-8 population was genotyped using the 5,385 SNP markers that covered seven linkage groups (LG). LG1 had the highest number (1,102) of SNPs and LG6 the lowest (439) (Table 4).

**Table 4:** Characteristics of the linkage groups of the LR-8 lentil population

**Linkage group**	**Length (cM)**	**Number of SNP markers**	**Number of SNP markers (%)**	**Average distance between markers (cM)**
**LG1**	151.8	1,102	20.5	0.13
**LG2**	175.2	676	12.5	0.25
**LG3**	167.7	940	17.4	0.17
**LG4**	169.2	835	15.5	0.20
**LG5**	102.5	849	15.8	0.12
**LG6**	117.9	439	8.1	0.26
**LG7**	88.8	544	10.1	0.16
**Total**	973.1	5,385		**Average:** 0.18

**Figure 2:**
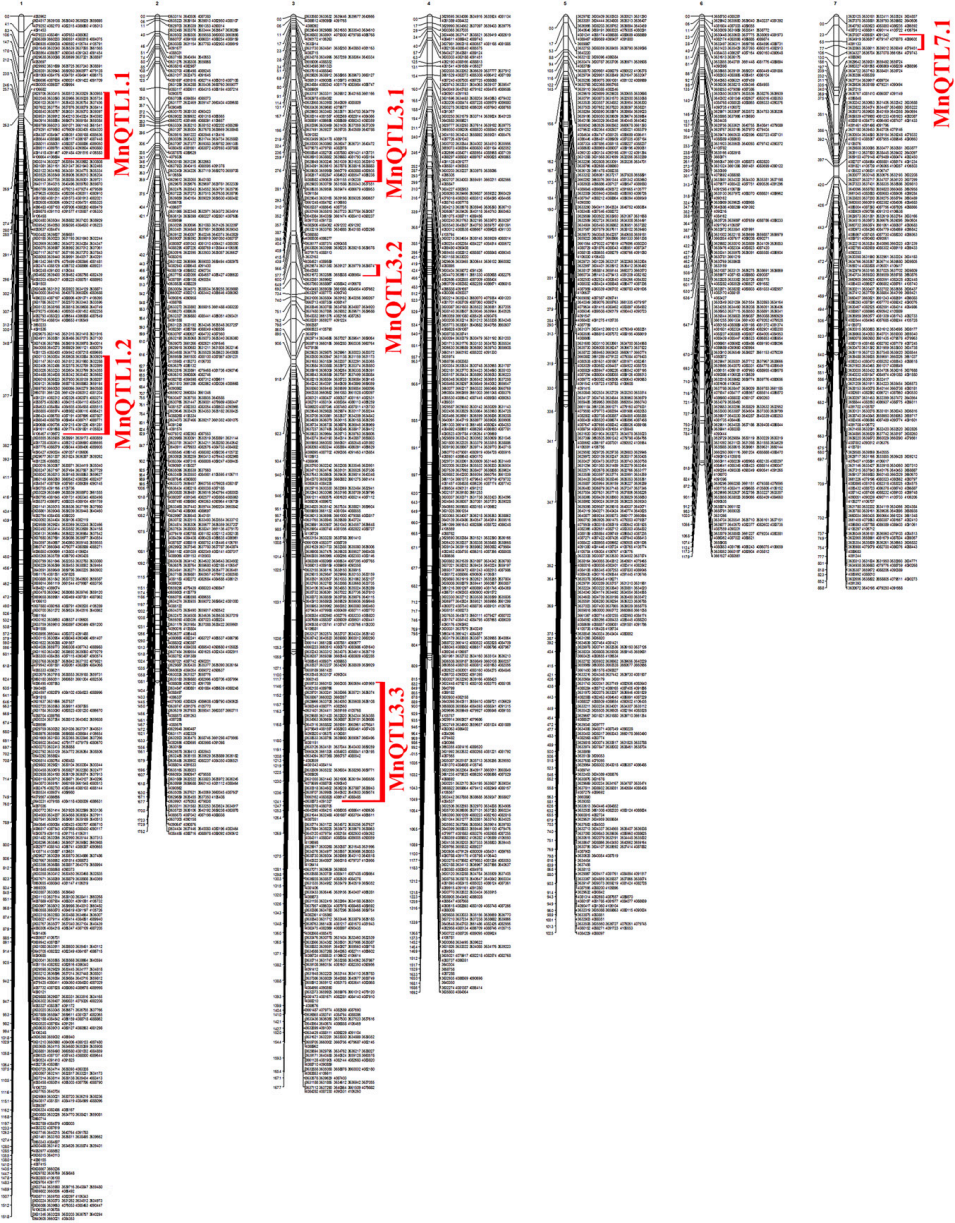
Genetic linkage map for lentil derived from the cross P1 × P2. Left bar of the LGs is cM and the right bar is marker names. QTL for Mn marked with red.

Linkage group size and average distance between adjacent markers are shown in Table 4. The smallest LG was LG7 (88.8 cM) and the largest was LG2 (175.2 cM). The total map length was 973.1 cM with an average distance of 0.18 cM between adjacent markers (Figure 2).

### QTL analysis of Mn

Six QTL, with LOD scores ranging from 3.02 to 4.42 and distributed across three linkage groups (LG1, LG3, and LG7) (Figure 2), were associated with seed Mn concentration and explained between 16.1 and 24.1% of the phenotypic variation (Table 5). The largest number of QTL regions for Mn concentration (3 QTL regions) were identified on LG3.

**Table 5:** Characteristics and locations of QTL regions for Mn concentrations in seeds of the LR-8 lentil population

**QTL region**	**LG**	**Position (cM)**	**Number of SNPs in the QTL region**	**% explanation**	Additive effect*	**LOD**	**Year/location**
**MnQTL1.1**	LG1	26.0-26.3	72	24.1	+	3.25	2012 Adana
**MnQTL1.2**	LG1	37.0-37.7	87	16.1	—	3.02	2012 Adana
**MnQTL3.1**	LG3	27.0-27.6	24	18.0	—	3.70	2013 Sanliurfa
**MnQTL3.2**	LG3	56.6-57.7	10	22.4	—	4.38	2012 Izmir; 2013 Izmir, Sanliurfa
**MnQTL3.3**	LG3	114.6-124.1	103	21.6	—	4.22	2012 Izmir, Sanliurfa; 2013 Izmir, Sanliurfa
**MnQTL7.1**	LG7	2.3-7.7	14	16.1	+	4.42	2012 Adana, Izmir, Sanliurfa; 2013 Adana, Izmir, Sanliurfa

* Positive (+) values of additive effect mean that the positive allele comes from parent P1, while negative (-) values mean that the positive allele comes from parent P2.

MnQTL3.1, located between 27.0-27.6 cM, was clustered with 24 SNP markers and explained 18.0% of the phenotypic variance. MnQTL3.2 and MnQTL3.3, located between 56.6-57.7 cM and 114.6-124.1 cM on LG3, explained 22.4 and 21.6% of the phenotypic variance, respectively. MnQTL3.2 clustered with 10 SNPs and MnQTL3.3 contained 103 SNPs. LG7 contained only one QTL region for Mn concentration. MnQTL7.1 was located between 2.3 and 7.7 cM on LG7 and explained 16.1% of the phenotypic variance. Additive effects of QTL regions of Mn are presented in Table 5.

## Discussion

Micronutrient malnutrition affects more than one-half of the total human population, with children and women at the highest risk (Ahmed *et al.* 2016). Biofortification aims to increase the total amount of minerals in the edible parts of crops by increasing the concentration of compounds, such as Mn, thus promoting their uptake by humans (Graham and Welch 1996). The biofortification strategy for alleviating this form of malnutrition is to increase the consistent daily intake of food staples by all family members, especially children and women, and to target the bridge between human nutrition and agriculture (Graham *et al.* 1999).

Mn accumulation in seed was determined to be quantitatively inherited in lentil. Supporting our results, previous QTL studies show that Mn concentration of seeds is quantitatively inherited in cabbage (Wu *et al.* 2008), *Lotus japonicus* (Klein and Grusak 2009), clover (Sankaran *et al.* 2009), canola (Ding *et al.* 2010), and Arabidopsis (Willems *et al.* 2010). Therefore, this study is also important with respect to understanding the genetic nature of Mn accumulation in seed. To date, no studies have identified QTL for Mn concentration in lentil. Identification of the QTL associated with high Mn concentration in lentil seeds could help select lines containing high Mn concentration in lentil breeding programs. This type of knowledge can be used to develop genetic strategies for molecular breeding to help increase the micronutrient content of edible parts of the lentil plant. Increased consumption of lentil with elevated levels of micronutrients could help to overcome micronutrient deficiency (Cichy *et al.* 2009), and the large variation in Mn concentration among the RILs could be the basis for developing such a strategy (Beebe *et al.* 2000).

### Mn variation

Mean Mn concentrations of seeds of RILs in the LR-8 population grown at three locations in 2 years varied from 8.5 to 26.8 mg/kg and represented a ∼threefold variation (Table 2). Previous reports indicate Mn concentrations range from 11.5 to 16.2 mg/kg for lentil landraces and from 11.5 to 15.4 mg/kg for lentil cultivars (Karaköy *et al.* 2012). Mn concentrations reported by Karaköy *et al.* (2012) are lower than those from the current study, which could be due to the different genotypes they used as well as different soil chemical properties of their experimental field. Per capita global lentil consumption is being increased rapidly and lentil fortification is a simple and promising approach to help decreased Mn deficiency (Podder *et al.* 2018). The data show that the Mn concentrations we observed could provide a significant amount of the required daily Mn from lentil in a given meal. For example, daily cooked lentil dal (50g/day) contains approximately 1 mg Mn (Mn concentrations in the current research found as a mean of 17.6 mg/kg, Table 2) which falls into recommended daily allowance (RDA) indicated by Santos *et al.* (2004) (0.7 to 22.0 mg for adults). In previous studies, Mn concentration was detected as a mean of 14 mg/kg in common bean seeds (Pinheiro *et al.* 2010). The Mn concentration was ranged between 9.2 -14.6 mg/kg in pea, between 4.4 and 12.6 mg/kg in buckwheat (Beitane and Krumina-Zemture 2017) and 16.8 mg/kg in seeds of chickpea (Kahraman *et al.* 2017). Mn value detected in the currrent study was higher as compared to other legumes.

The ANOVA for Mn concentration shows that location, year, and genotype interactions are statistically significant (Table 3). Interactions among genotypes, locations, and years are likely due to different environmental conditions affecting the availability of Mn in the pool of soil micronutrients available for plant uptake (Sankaran *et al.* 2009).

### Linkage mapping

DArT analysis allowed the construction of high-density linkage maps with a very large number of SNPs. In the current study, the DArT method generated 10,552 SNPs. Using this DArT approach on the parental RIL populations, a total of 5,385 SNPs were mapped (Table 4). The amount of data used for mapping purposes was similar to previous DArT analysis studies (Poland *et al.* 2012; Li *et al.* 2014; Han *et al.* 2016).

In this study, the linkage map of lentil consisted of seven linkage groups with 5,385 SNP markers. The total map length (973.1 cM) in the current study is shorter than for many previous lentil mapping studies, *e.g.*, 1,073 cM (Eujayl *et al.* 1997), 1,868 cM (Tullu *et al.* 2008), 1,396.3 cM (Tanyolac *et al.* 2010), 3,843 cM (Gupta *et al.* 2012), and 834.7 cM (Sharpe *et al.* 2013). Recently, the lentil genome was mapped with 1,784 markers (including SNP and SSR) covering a genome size of 4,060.6 cM using genotype by sequencing (GBS) in RILs developed from “PI 320937” × “Eston” parents (Ates *et al.* 2016). On the other hand, a total map length of 784.1 cM, which is close to our map length, was detected using a few markers [100 Random Amplified Polymorphic DNA (RAPDs), 11 Inter Simple Sequence Repeats (ISSRs) and 3 Resistance Gene Analogs (RGAs)] (Rubeena *et al.* 2003). Another study constructed a genetic linkage map using 6 SSRs and 537 contigs covering a genome size of 834.7 cM (Sharpe *et al.* 2013). Overall, the genetic map in this study is more robust compared to previous QTL mapping studies in lentil (Eujayl *et al.* 1997; Tullu *et al.* 2008; Tanyolac *et al.* 2010; Gupta *et al.* 2012; Sharpe *et al.* 2013).

The seven major LGs constructed in the current study correspond to the seven haploid chromosome number of lentil (Sharpe *et al.* 2013; Ates *et al.* 2016). Differences in the estimated distances of both parental maps may reflect differences in the recombination frequencies of both parents. Putative causes for the difference between the two estimated parental genome maps include marker distribution along the chromosome that varies between parents, and male and female gametes that probably display different recombination frequencies (Khadari *et al.* 2010).

### QTL analysis of Mn

This study is the first to map QTL for Mn concentration in lentil seeds and uses a larger number of SNPs than previous studies mapping the lentil genome. P1 (CDC Redberry) is adapted to the northern temperate zone, and was the parent that had the highest seed Mn concentration. A total of 6 QTL for Mn concentration in seeds of the LR-8 population were identified using CIM. These were distributed across three linkage groups in the LR-8 lentil population (Table 5).

In a study of QTL associated with Mn concentration in *Lotus japonicus* (Klein and Grusak 2009) two QTL explaining 35.2% of the phenotypic variation were identified on chromosomes 1 and 2. Ten QTL for Mn concentration were distributed across 8 chromosomes, with LOD scores ranging between 3.34-6.55, explaining between 9.06 and 16.43% of the phenotypic variation in *Brassica napa* (Ding *et al.* 2010). In other similar studies, six QTL for Mn concentration were found in one of two wheat populations (Pu *et al.* 2013), two QTL were mapped in soybean (Ramamurthy *et al.* 2014), and four QTL associated with Mn concentration were identified in rice (Yu *et al.* 2015). Here, the number of QTL detected was high; micronutrient accumulation in seeds continues to be a complex process controlled by poly-genes (Grusak and DellaPenna 1999).

For Mn in lentil seeds, six QTL were statistically significant, and the phenotypic variation ranged from 16.1 to 24.1% with LOD scores of 3.02-4.42. QTL analysis of nutrient element accumulation in seeds of other crops shows that the value for explaining phenotypic variation typically ranges between 9.06 and 35.2% (Ding *et al.* 2010; Klein and Grusak 2009; Yu *et al.* 2015). The value we found falls within the same range, and our estimates of phenotypic variation of seed Mn concentration in lentil were similar to those for canola (Ding *et al.* 2010) and wheat (Pu *et al.* 2013).

### Conclusions

The LR-8 lentil population studied here demonstrated large phenotypic variation in terms of Mn concentrations in seeds. Mn concentrations in lentil seeds were observed to be quantitatively inherited. DArT analysis allowed the construction of high-density linkage maps with a large number of SNPs. The QTL that were stable across 2 years and three locations were unaffected by environmental conditions, and therefore could be used in marker-assisted selection in lentil breeding programs. We believe that this work is the first to map QTL for Mn concentrations in lentil seeds. The discovery of QTL for seed Mn concentration could have significant implications for biofortification breeding strategies. The QTL analysis might help to resolve some of the complexity with respect to Mn accumulation in lentil grain. RIL LR8-113, which contained the highest Mn concentration, could be used as a parent in breeding programs. The results of this study can be applied to the development of lentil genotypes with higher Mn concentrations. The high-density maps could increase fundamental knowledge of the genome structure of lentil, help in future construction of physical maps, and serve as a basis for map-based cloning in lentil.

## Supplementary Material

Supplemental material is available online at www.g3journal.org/lookup/suppl/doi:10.1534/g3.118.200259/-/DC1.
